# Asialoglycoprotein Receptor-Targeted Superparamagnetic Perfluorooctylbromide Nanoparticles

**DOI:** 10.1155/2021/5510071

**Published:** 2021-05-29

**Authors:** Yang Li, Chao Feng Yang, Hui Zuo, Ao Li, Sushant Kumar Das, Jin Hong Yu

**Affiliations:** ^1^Department of Radiology, The Affiliated Hospital of North Sichuan Medical College, 63 Wenhua Road, Nanchong City, Sichuan Province 637000, China; ^2^Department of Ultrasound, The Affiliated Hospital of North Sichuan Medical College, 63 Wenhua Road, Nanchong City, Sichuan Province 637000, China; ^3^Department of Ultrasound, The First Affiliated Hospital of Nanjing Medical University, Nanjing City, Jiangsu Province 210000, China; ^4^Department of Ultrasound, The People's Hospital of Yuechi County, 22 East Jianshe Road, Yuechi County, Sichuan Province 638350, China

## Abstract

**Background:**

The decrease in asialoglycoprotein receptor (ASGPR) levels is observed in patients with chronic liver disease and liver tumor. The aim of our study was to develop ASGPR-targeted superparamagnetic perfluorooctylbromide nanoparticles (M-PFONP) and wonder whether this composite agent could target buffalo rat liver (BRL) cells in vitro and could improve *R*2^*∗*^ value of the rat liver parenchyma after its injection in vivo.

**Methods:**

GalPLL, a ligand of ASGPR, was synthesized by reductive amination. ASGPR-targeted M-PFOBNP was prepared by a film hydration method coupled with sonication. Several analytical methods were used to investigate the characterization and safety of the contrast agent in vitro. The in vivo MR *T*2^*∗*^ mapping was performed to evaluate the enhancement effect in rat liver.

**Results:**

The optimum concentration of Fe_3_O_4_ nanoparticles inclusion in GalPLL/M-PFOBNP was about 52.79 *µ*g/mL, and the mean size was 285.6 ± 4.6 nm. The specificity of GalPLL/M-PFOBNP for ASGPR was confirmed by incubation experiment with fluorescence microscopy. The methyl thiazolyl tetrazolium (MTT) test showed that there was no significant difference in the optical density (OD) of cells incubated with all GalPLL/M-PFOBNP concentrations. Compared with M-PFOBNP, the increase in *R*2^*∗*^ value of the rat liver parenchyma after GalPLL/M-PFOBNP injection was higher.

**Conclusions:**

GalPLL/M-PFOBNP may potentially serve as a liver-targeted contrast agent for MR receptor imaging.

## 1. Introduction

The asialoglycoprotein receptor (ASGPR) is a hepatocyte-specific receptor and mediates the rapid clearance of asialoglycoproteins from the circulation [1, 2]. Although the primary physiological role of ASGPR is considered to be the clearance of the circulation of glycoproteins that contain terminal galactose or N-acetylglucosamine residues, a lot of other physiologic roles, such as removal of apoptotic cells, fibronectin, and immunoglobulin A, have been reported [3–5]. The ASGPR is predominantly expressed on the sinusoidal surface of the hepatocytes. A significant reduction in ASGPR expression is observed in patients with viral hepatitis, cirrhosis, and hepatocellular carcinoma [6, 7]. The decrease in ASGPR levels can result in the retention of fibronectin, leading to the progression of liver fibrosis [8]. Moreover, the accumulation of apoptotic cells might induce proinflammatory and profibrogenic responses. Therefore, the evaluation of ASGPR can provide considerable information on the pathobiologic changes of liver injure.

In recent years, molecular image has emerged as an imaging strategy that combines advanced image technology with targeted contrast agents for evaluating biological processes at the molecular level. Molecular image is a novel tool that has allowed noninvasive diagnostic imaging as well as semiquantitative and quantitative evaluation of target expression. Recent advancements of nanotechnology have extended molecular image research to include magnetic resonance (MR) imaging and ultrasound (US).

A variety of nanoparticle platforms have been developed for targeted molecular imaging and drug delivery. The perfluorocarbon (PFC) nanoparticle is a unique platform technology which may be applied to clinically relevant modalities [9]. PFC nanoparticles show a highly stable, biologically inert, and nontoxic platform that can be designed to accomplish a variety of molecular imaging and drug delivery functions in vivo. PFC nanoparticles are approximately 250 nm in diameter and consist of a liquid PFC core encapsulated within a monolayer of phospholipids which provides an ideal surface decorated with targeting ligands, imaging agents, and drugs either individually or in combination [9–11]. The targeting ligands and imaging agents are coupled to decorated phospholipids allowing for controlling location of these compounds to target the surrounding biological environment [11]. These targeted agents via intravenous injection accumulate at intended sites overexpressing specific biological markers. Different PFC cores can be used, including perfluorodecalin, perfluorodichlorooctane, and most commonly perfluorooctylbromide (PFOB) [12].

Superparamagnetic iron oxide (SPIO) nanoparticles can be considered as a negative MR contrast agent (*T*2 agent), which exhibit interesting properties such as superparamagnetism, high saturation field, high field irreversibility, and extra anisotropy contributions [13]. SPIO nanoparticles constitute specific iron oxide cores which are coated with hydrophilic agents, e.g., polymers, oleic acid, dextrans, starch, etc. [9]. Moreover, SPIO nanoparticles have a high magnetic susceptibility and produce local disturbances in the field which result in loss of signal through *T*2^*∗*^ = (1/*R*2^*∗*^) decay, especially on gradient echo images [9].

It is noteworthy that our research group combined the advantages of PFOB nanoparticles and Fe_3_O_4_ nanoparticles to prepare a composite agent superparamagnetic PFOB nanoparticles (M-PFOBNP) which may potentially use as a multimodal contrast agent for enhanced MR, US, and computed tomography (CT) imaging [14]. Herein, we would like to functionalize M-PFOBNP with targeting ligands and make it become a multimodality and multifunctional imaging agent.

Ligands recognized by the ASGPR include glycoproteins containing terminal galactose or N-galactosamine residues. In the present study, we prepared galactosylated poly-L-lysine (GalPLL)/M-PFOBNP and explored whether this contrast agent could target BRL cells in vitro and could improve *R*2^*∗*^ value of the rat liver parenchyma after administration of the contrast agent in vivo.

## 2. Materials and Methods

### 2.1. Preparation of GalPLL/M-PFOBNP

GalPLL was prepared by using reductive amination. The galactose (Sigma-Aldrich, St. Louis, MO, USA) and poly-L-lysine (Sigma-Aldrich) at the weight ratio of 1 : 3 were dissolved in 10 mL phosphate buffered saline (PBS). After addition of 1 mg sodium borohydride (NaBH_4_) the reaction mixture was incubated at room temperature for 24 h, and purified on Sephadex G-25 column.

The M-PFOBNP was prepared using a method reported by Li et al. with minor modifications [14]. Briefly, the agent consisted of liquid PFOB (Elf Atochem, Paris, France), oleic acid-treated Fe_3_O_4_ nanoparticles (Size 10 nm, Ocean NanoTech Inc., Arkansas, USA), and a surfactant comixture (Avanti Polar Lipids Inc., AL, USA) including 70 mol% egg yolk lecithin, 29 mol% cholesterol, and 1 mol% 1, 2-distearoyl-sn-glycero-3-phosphoethanolamine-N-[amino (polyethylene glycol)2000]. The surfactant commixture and varying volumes of Fe_3_O_4_ nanoparticles were dissolved in chloroform, evaporated under reduced pressure, and formed to a dry lipid film under vacuum. For targeted agent, 5 mL GalPLL solution was added to the dry lipid film. The liposome suspension was combined with liquid PFOB (30% *v*/*v*), and was emulsified for 4 min by an XL2020 sonicator (Heat Systems Inc, NJ, USA). The free Fe_3_O_4_ nanoparticles in the emulsion were removed by using the magnetic isolation method [15]. The final sample was stored in a freezer at 4°C. M-PFOBNP was prepared similarly with the addition of 5 mL PBS instead of 5 mL GalPLL solution.

### 2.2. Characterization of GalPLL/M-PFOBNP

The concentration of Fe_3_O_4_ nanoparticles in the emulsion was measured by an atomic absorption spectrophotometer (AAS, Hitachi Z-5000, Tokyo, Japan).

GalPLL/M-PFOBNP with different concentration of Fe_3_O_4_ nanoparticles were placed in Eppendorf tubes of 1 cm in diameter. For *T*2^*∗*^-weighted imaging, an axial multi-echo fast gradient echo sequence with 6 echoes was performed. The parameters were as follows: TR/TE range, 160/2.7–22.3 ms; slice thickness, 2.5 mm; flip angle 30°. The transverse relaxation rate *R*2^*∗*^ (1/*T*2^*∗*^) was measured. All MR studies were performed with a 3-T system (Discovery 750, GE Healthcare, Milwaukee, Wisconsin, USA).

The mean size of GalPLL/M-PFOBNP was determined with a laser light-scattering submicron particle sizer (Malvern Instruments, Malvern, Worcestershire, United Kingdom).

### 2.3. Cells and Cell Culture

BRL cells were purchased from Harry bioengineering Co., Ltd. (Sichuan, China) and were incubated with Rosewell Park Memorial Institute (RPMI)-1640 medium (Gibco, Manchester, UK) containing 10% fetal bovine serum (FBS) at 37°C in a humid incubator with 5% CO_2_.

### 2.4. Cytotoxicity of GalPLL/M-PFOBNP

Cell viability was estimated using the MTT assay. BRL cells were seeded into 96-well plates at 2 × 10^3^ per well 24 h before incubation for 3 h with different volume fractions (1%, 5%, 10%, and 15%) of GalPLL/M-PFOBNP (52.79 *µ*g/mL Fe_3_O_4_ concentration) solution that were diluted by the addition of RPMI-1640 medium containing 10% FBS. Cells in culture medium without GalPLL/M-PFOBNP were used as a control. Cell viability was measured by the addition of 20 *μ*L MTT solution (5 mg/mL, Sigma, St. Louis, MO, USA) to each well for 4 h incubation. Then, 150 *μ*L dimethyl sulfoxide (Sigma, St. Louis, MO, USA) was added to dissolve the formazan crystals formed. To assess cell viability, OD was calculated at 490 nm using an enzyme-linked immunosorbent assay plate reader.

### 2.5. Target Assay

BRL cells were seeded at an initial density of 1 × 10^5^ cells/mL in 6-well cell culture plate for 24 h. 5 *μ*L DiI was added to each well for labeling cells. DiI is a lipophilic membrane dye that diffuses into the cell membrane and gradually stains the entire cell membrane. The cells were incubated for 15 min, and then the medium was aspirated and the cells were washed five times with PBS. Afterwards, the cells were incubated with fresh medium containing the same volume of fluorescein isothiocyanate- (FITC-) labeled GalPLL/M-PFOBNP (52.79 *µ*g/mL Fe_3_O_4_ concentration) for 0.5, 1, 1.5, and 2 h. Cells in culture medium with FITC-labeled M-PFOBNP were used as a control. The combination of GalPLL/M-PFOBNP and BRL cells at different time points was estimated by a fluorescence microscope (Olympus IX71, Tokyo, Japan).

### 2.6. Animal Preparation

Animal experiments were approved by our animal ethics committee. Sixteen male Sprague-Dawley rats (200–250 g, Laboratory Animal Center of North Sichuan Medical College, Nanchong, China) were randomized into two groups of ten rats each. One group was injected with GalPLL/M-PFOBNP (52.79 *µ*g/mL Fe_3_O_4_ concentration). As a control, the other group was injected with M-PFOBNP. The two kinds of contrast agents were administered into the caudal vein at a dose of 2 mL/kg body weight. Anesthesia was induced by intraperitoneal injection of 3% pentobarbital sodium (1 mL/kg).

### 2.7. In Vivo MR Imaging

After anaesthesia, animals were positioned prone. MR imaging was accomplished through a 3-T system (Discovery 750, GE Healthcare, Milwaukee, Wisconsin, USA) using a 3-inch-diameter circular surface coil. The liver images were obtained before, at 30 s, 0.5 h, 1 h, 2 h, 4 h, 8 h, 12 h, and 24 h after the injection of GalPLL/M-PFOBNP or M-PFOBNP. For the quantification of liver *R*2^*∗*^ values, images were obtained using an axial multi-echo fast gradient echo sequence with 6 echoes (TR/TE range, 160/2.7–22.3 ms; slice thickness, 2.5 mm; flip angle 30°).

The software of *R*2Star (Function tool 4.4, GE Healthcare, Milwaukee, Wisconsin, USA) was used for calculating the *R*2^*∗*^ values. Two junior radiologists with 3-year experience in abdominal MRI analyzed the same two consecutive *R*2^*∗*^ slices at mid liver. Three regions-of-interest (ROIs) of approximately 2-3 mm^2^ were placed on each slice, avoiding major blood vessels and artifacts. Mean *R*2^*∗*^ values for all six ROIs in each liver were recorded, and values were averaged for all six ROIs in each liver.

### 2.8. Biodistribution Analysis of the GalPLL/M-PFOBNP

After the MR examinations, the animals were sacrificed by intraperitoneal injection of 3% pentobarbital sodium (3.0 ml/kg). The iron concentration in various organs (liver, spleen, kidneys, and brain) was determined quantitatively by AAS.

### 2.9. Statistical Analysis

Data were expressed as mean ± SD (standard deviation). Statistical analyses were performed using SPSS software (SPSS Inc., Chicago, IL, USA). Statistical differences for experimental groups were evaluated using a one-way ANOVA and Student's *t*-test. A *P* value ˂0.05 was considered statistically significant.

## 3. Results

### 3.1. Characterization of GalPLL/M-PFOBNP

The concentration of Fe_3_O_4_ nanoparticles in the emulsion measured by atomic absorption spectrophotometer was 8.04, 14.54, 28.61, 40.65, and 52.79 *µ*g/mL, respectively.

**Figure 1:**
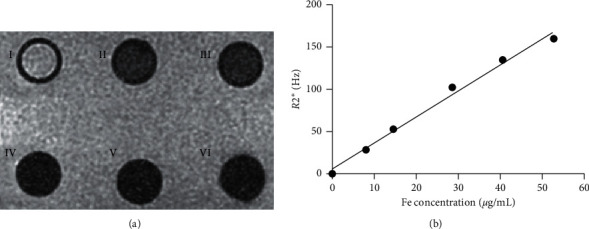
In vitro MR image. (a) I: PBS, II-VI: GalPLL/M-PFOBNP with different Fe_3_O_4_ concentrations (II-VI are 8.04, 14.54, 28.61, 40.65, and 52.79 *µ*g/mL, respectively). (b) The linear fit was obtained between the Fe_3_O_4_-inclusion concentration in the GalPLL/M-PFOBNP and the transverse relaxation rate *R*2^*∗*^.

The transverse relaxation rate *R*2^*∗*^ (1/*T*2^*∗*^) was measured by using 3-T MR scanner. Figure 1(a) includes *T*2^*∗*^-weighted MR images of GalPLL/M-PFOBNP samples with different Fe_3_O_4_ concentrations (Figure 1(a)). The relationship between the transverse relaxation rate *R*2^*∗*^ value and the Fe_3_O_4_ nanoparticle concentration can be fitted to a linear function (*r* = 0.962) (Figure 1(b)).

**Figure 2:**
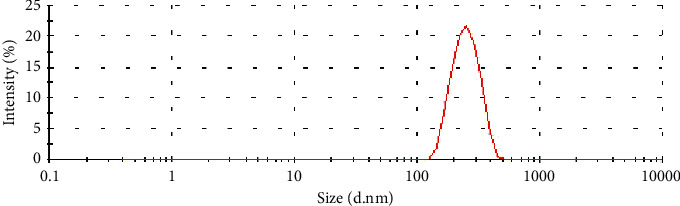
The size distributions of GalPLL/M-PFOBNP by dynamic light-scattering measurement.

The size distribution of varying concentration of Fe_3_O_4_ nanoparticles was in the range of 250–300 nm. The mean diameter of GalPLL/M-PFOBNP (52.79 *µ*g/mL Fe_3_O_4_ concentration) was 285.6 ± 4.6 nm and the size distribution was nearly symmetric (Figure 2).

### 3.2. Cytotoxicity

The OD of BRL cells incubated with 1%, 5%, 10%, or 15% *v*/*v* GalPLL/M-PFOBNP solution were 0.300 ± 0.011, 0.297 ± 0.010, 0.296 ± 0.008, and 0.295 ± 0.009, respectively. The OD of the control group was 0.301 ± 0.010. The MTT assay indicated that there was no significant difference on the OD of BRL cells incubated with all GalPLL/M-PFOBNP concentrations (*P* > 0.05).

### 3.3. The Combination of GalPLL/M-PFOBNP and BRL Cells

**Figure 3:**
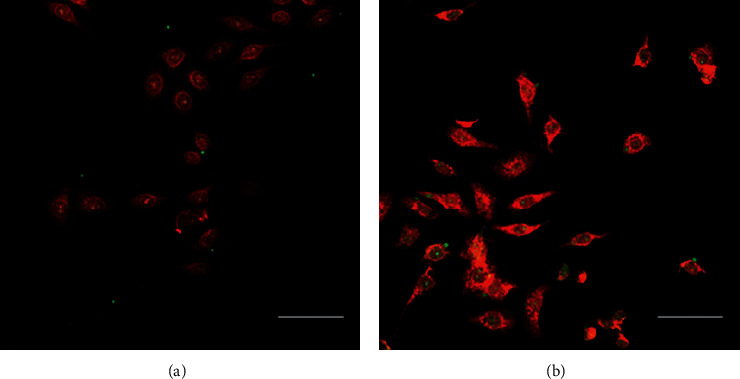
The combination of GalPLL/M-PFOBNP and BRL cells was observed under fluorescence microscopy. BRL cells (red) after incubation with FITC-labeled GalPLL/M-PFOBNP (green) for 0.5 h (a) and 1 h (b), respectively. Scale bar: 10 *μ*m.

After DiI staining, the plasmalemma of the BRL cells appeared red and FITC-labeled GalPLL/M-PFOBNP appeared green under fluorescence microscopy. After 0.5 h of application with fresh medium containing the same volume of FITC-labeled GalPLL/M-PFOBNP, a few FITC-labeled GalPLL/M-PFOBNP conglutinated to the BRL cells (Figure 3(a)). A large number of GalPLL/M-PFOBNP combined with BRL cells were observed after 1 h (Figure 3(b)). There was no significant increase in the combination of FITC-labeled GalPLL/M-PFOBNP and BRL cells in 1.5 and 2 h, respectively. However, we could not observe that the FITC-labeled M-PFOBNP conglutinated to BRL cells.

### 3.4. In Vivo Rat Liver MR Imaging

**Figure 4:**
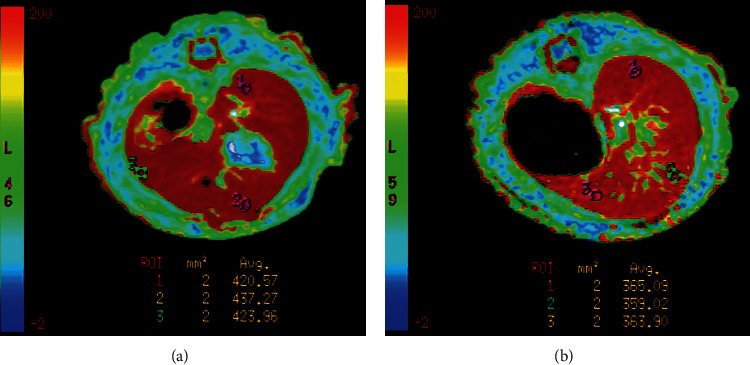
MR *R*2*∗* maps of the rat liver at 2 h after injection of GalPLL/M-PFOBNP (a) and M-PFOBNP (b), respectively. The increase in *R*2*∗* value of the rat liver parenchyma after administration of GalPLL/M-PFOBNP is higher than that after administration of M-PFOBNP.

**Figure 5:**
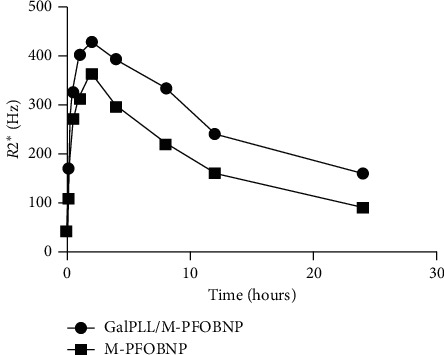
The relationship between *R*2*∗* value and time after administration of different contrast agents.

The mean *R*2^*∗*^ value before administration of agents was 41.97 ± 2.33 Hz and 42.24 ± 3.30 Hz, respectively, in GalPLL/M-PFOBNP group and M-PFOBNP group (*P* > 0.05). Serial measurement of *R*2^*∗*^ value revealed that the maximal *R*2^*∗*^ value of rat liver was observed at approximately 2 h after administration of two agents and 427.75 ± 7.39 Hz and 363.31 ± 4.73 Hz, respectively, in GalPLL/M-PFOBNP group and M-PFOBNP group (*P* < 0.05) (Table 1) (Figures 4 and 5). However, dramatic increase of the *R*2^*∗*^ value of entire hepatic parenchyma was detected on *R*2^*∗*^ map images enhanced with GalPLL/M-PFOBNP, and the peak enhancement of liver in the group with GalPLL/M-PFOBNP was much greater than those in the group with M-PFOBNP (*P* < 0.001) (Table 1) (Figure 5). The elimination of GalPLL/M-PFOBNP in the liver was significantly slower than that of M-PFOBNP (Figure 5).

### 3.5. Biodistribution Assessment

At 24 hours post dose, the iron concentration in various organs is shown in Table 2. 96.38 ± 4.69 *μ*g Fe/g of the iron concentration reached in the liver following a dose of 2 mL/kg IV dose of the GalPLL/M-PFOBNP, compared to 88.13 ± 3.60 *μ*g Fe/g of the M-PFOBNP (*P*=0.001). However, the iron concentration in spleen, kidneys, and brain has no significant differences.

## 4. Discussion

In the present study, we have established that GalPLL/M-PFOBNP can be targeted specifically to ASGPR on hepatocytes. The findings are supported by target assay in vitro and MR imaging data in vivo. GalPLL/M-PFOBNP, an ASGPR-targeted MR contrast agent, can be used to study ASGPR biology in intact animals. This agent is M-PFOBNP that has been conjugated with GalPLL.

Glycoproteins containing terminal galactose or N-galactosamine residues are ligands for the ASGPR. GalPLL was used to mediate specific gene transfer into hepatocytes. The receptor-mediated targeting of plasmid DNA to hepatocytes was achieved through a plasmid DNA-GalPLL complex [16]. GalPLL-antisense oligodeoxynucleotides (asODNs) as an anti-HBV agent can be absorbed by hepatocytic cells and concentrated in liver for efficiently blocking HBV gene expression [17]. Demetz et al. [18] reported that small hairpin RNA plasmid specific for rabbit scavenger receptor-class B type I (SR-BI) was complexed with GalPLL, achieving an organ-selective, receptor-mediated gene transfer. In the present study, GalPLL was synthesized by reductive amination, conjugating the phospholipid shell of M-PFOBNP as the targetable ligand to hepatocytes.

Molecular imaging is expected to play an important role in the early diagnosis of diseases, disease staging, and disease monitoring in the future [19]. Nowadays, macroscopic imaging systems that provide anatomical and limited physiological information are in widespread use. Such systems include MR, US, and CT [19]. With the development of targeted contrast agents for those systems, the spectrum of clinical molecular imaging applications is expanding. The ASGPR is hepatocyte specific and responsible for rapid clearance of asialoglycoproteins from the circulation by receptor-mediated endocytosis. The discovery of the ASGPR system has resulted in the advance of diagnostic and therapeutic agents. A reduction in ASGPR content is a correlation with the progression of liver injury and the presence of liver tumor [20–22]. The production of specific site-targeted contrast agents remains a goal for medical imaging systems. Contrast agents decorated with binding ligands improve their binding affinity to target to specific molecular markers, and decrease the rate of dissociation. The specific interaction between asialoglycoproteins and the ASGPR was exploited in the development of a receptor-targeted contrast agent for the liver image. Weissleder et al. [23] reported that ultrasmall superparamagnetic iron oxide (USPIO) particles were directed to ASGPR by binding galactose terminals in the form of arabinogalactan (AG) to these particles. Biodistribution data showed AG-USPIO particles selectively accumulate in the liver but not in other organs. The signal intensity of the liver after contrast agent injection was lower in rats injected with AG-USPIO than in rats that had received USPIO. Leveille-Webster et al. [24] demonstrated that a novel ASGPR-directed hepatic MR contrast agent, BMS 180550, can provide information on ASGPR function in normal rats and suggested that this agent may be used to monitor regenerative responses after acute liver injury or resection. Huang et al. [25] reported that galactose-terminal-amino-functionalized SPIO (Gal-ASPIO) can be targeted specifically to hepatocytes by ASGPR on cell surfaces. Furthermore, the association of Gal-ASPIO to HepG2 cells was reduced by free galactose, supporting the model of ASGPR-mediated binding.

The liquid PFC core is surrounded by a phospholipid monolayer which can be functionalized with a wide variety of agents including homing ligands, drugs, and imaging agents. Our coworkers took advantage of PFOB and Fe_3_O_4_ to prepare M-PFOBNP and found that M-PFOBNP caused higher echogenicity than PFOB nanoparticles and had strong magnetic susceptibility and radiopacity as a multimodal contrast agent in our previous study [14]. These findings underlined the potential application of GalPLL/M-PFOBNP as a multimodality and multifunctional contrast agent. In the present study, the maximal *R*2^*∗*^ value of the liver after administration of contrast agent was higher in rats injected with GalPLL/M-PFOBNP than in rats that had received M-PFOBNP (427.75 ± 7.39 Hz compared with 363.31 ± 4.73 Hz; *P* < 0.05). At 24 h post injection, the iron concentration in the liver was 96.38 ± 4.69 *μ*g Fe/g and 88.13 ± 3.60 *μ*g Fe/g, respectively (*P*=0.001). Therefore, the target-mediated process increased the level of iron in the liver in comparison to M-PFOBNP. Attachment to GalPLL targets biodistribution of these particles exclusively to hepatocytes. GalPLL/M-PFOBNP has several advantages over the use of M-PFOBNP. First of all, because the majority of the dose accumulates in the liver, the injection dose can be decreased. The maximal *R*2^*∗*^ value of rat liver was observed at approximately 2 h. Secondly, the molecular imaging of GalPLL/M-PFOBNP may provide a tool to evaluate the change of ASGPR function by imaging signal and a quantitative in vivo measurement of ASGPR function that may be clinically useful for monitoring disease states and therapeutic response. Lastly, because most malignant tumors are deficient in ASGPR, GalPLL/M-PFOBNP is expected to provide excellent contrast between normal liver and metastases. This receptor-targeted contrast agent is different from other hepatic MR contrast agents that are phagocytosed by mononuclear cells and whose hepatic selectivity is based on the fact that Kupffer cells take responsibility for the mononuclear phagocytic activity [26].

In conclusion, our study demonstrates in vivo MR imaging with a novel, ASGPR-targeted contrast agent that is used to improve *R*2^*∗*^ value of the rat liver parenchyma after its injection and may provide information on ASGPR function. GalPLL/M-PFOBNP can be used as a multimodality and multifunctional contrast agent for further research.

## Figures and Tables

**Table 1:** The *R*2*∗* value (Hz) changes before and 2 h after the injection of different contrast agents.

Group	Precontrast	Postcontrast	*P* value
GalPLL/M-PFOBNP	41.97 ± 2.33^▲^	427.75 ± 7.39^★^	<0.001
M-PFOBNP	42.24 ± 3.30^▲^	363.31 ± 4.73^★^	<0.001

^▲^ *P* > 0.05 compared with *R*2*∗* value of the precontrast. ^★^*P* < 0.001 compared with *R*2*∗* value of the postcontrast.

**Table 2:** Biodistribution of different contrast agents determined by AAS iron measurements (*μ*g Fe/g) in four main organs.

Organ	GalPLL/M-PFOBNP	M-PFOBNP	*P* value
Liver	96.38 ± 4.69	88.13 ± 3.60	0.001
Spleen	31.50 ± 4.24	31.75 ± 3.99	0.905
Brain	10.38 ± 1.69	9.50 ± 1.93	0.350
Kidney	20.13 ± 3.30	19.38 ± 2.26	0.605

## Data Availability

The data that support the findings of this study are available from the corresponding author upon reasonable request.
